# Lupus-like Disease in *FcγRIIB*^−*/*−^ Mice Induces Osteopenia

**DOI:** 10.1038/s41598-019-53963-z

**Published:** 2019-11-22

**Authors:** Peerapat Visitchanakun, Worasit Saiworn, Prapaporn Jongwattanapisan, Asada Leelahavanichkul, Prapaporn Pisitkun, Sutada Lotinun

**Affiliations:** 1 Department of Physiology, Faculty of Dentistry, Chulalongkorn University, Bangkok, Thailand; 2 Skeletal Disorders Research Unit, Faculty of Dentistry, Chulalongkorn University, Bangkok, Thailand; 3 Department of Veterinary Medicine, Faculty of Veterinary Science, Chulalongkorn University, Bangkok, Thailand; 4 Division of Immunology, Department of Microbiology, Faculty of Medicine, Chulalongkorn University, Bangkok, Thailand; 5 Division of Allergy, Immunology, and Rheumatology, Department of Medicine, Faculty of Medicine, Ramathibodi Hospital, Mahidol University, Bangkok, Thailand

**Keywords:** Bone quality and biomechanics, Lupus nephritis

## Abstract

Osteoporotic fracture is a major cause of morbidity in patients with systemic lupus erythematosus (SLE). Mice lacking Fc gamma receptor IIb (*FcγRIIB*) spontaneously develop lupus-like disease or SLE at 6-month-old. The aim of this study was to investigate whether *FcγRIIB* deletion induces osteopenia. μCT analysis indicated that deleting *FcγRIIB* did not affect cancellous bone microarchitecture in 3-month-old mice in which SLE had not yet developed. However, 6- and 10-month-old *FcγRIIB*^−*/*−^ males that developed an SLE-like phenotype were osteopenic and *FcγRIIB* deletion resulted in decreased cancellous bone volume. Histomorphometry confirmed a significant decrease in cancellous bone volume in 6- and 10-month-old *FcγRIIB*^−*/*−^ males. The osteoclast number was increased without any change in osteoblast number. *In vitro* assays indicated that deleting *FcγRIIB* increased osteoclast differentiation while alkaline phosphatase activity and mineralization were unaltered. These changes were associated with increases in steady-state mRNA levels for the osteoclast marker genes *Trap* and *Ctsk*. Moreover, *FcγRIIB*^−*/*−^ mice had higher level of serum TNFα, a proinflammatory cytokine. A soluble TNFα receptor, etanercept, prevented cancellous bone loss in *FcγRIIB*^−*/*−^ mice. Our results indicate that *FcγRIIB* indirectly regulates cancellous bone homeostasis following SLE development. *FcγRIIB* deletion induces inflammatory bone loss due to increased TNFα-mediated bone resorption without any change in bone formation in mice with SLE-like syndrome.

## Introduction

Systemic autoimmune diseases with complex multifactorial etiologies including systemic lupus erythematosus (SLE) are associated with low bone mass and fracture. SLE is more common in African American, Hispanic, and Asian compared to Caucasian women. Patients with SLE are at an increased risk for osteoporosis for several reasons. Systemic inflammation, metabolic factors, serological factors, hormonal factors, genetic factors and medication can increase bone loss in these patients^1^. Glucocorticoids commonly prescribed for SLE because of their rapid and board spectrum in suppressing disease activity and preventing irreversible organ damage can trigger significant bone loss. However, it has been shown that SLE patients on long-term glucocorticoids and those not on glucocorticoids have significantly decreased BMD compared to healthy individuals^2^. There was no difference in BMD between the patients taking glucocorticoids and no glucocorticoids. Therefore, SLE itself or other factors may have deleterious effects on bone mass.

Osteoimmunology studies indicate a complex interplay between the immune and skeleton systems. Although osteoporosis and high fracture risk are well-known consequences of SLE, the cause of low bone mass in SLE patients remains unclear. The deposition of immune complexes (ICs) plays a major role in the pathogenesis of SLE. Receptors for the Fc domain of IgG (FcγRs) are important for IC clearance. In SLE, impaired Fc-mediated IC clearance initiates the release of inflammatory mediators and influx of inflammatory cells. Four classes of FcγR, FcγRI, FcγRII, FcγRIII and FcγRIV have been identified in mice^3^. FcγRIIB, a negative regulator of IC-triggered activation, is associated with susceptibility to autoimmune disease, particularly SLE^4,5^. This inhibitory receptor functions to suppress the development of autoimmunity by regulating B-cell responses and effector cell activation^6^. The abnormal low expression of B-cell *FcγRIIB* in SLE leads to inadequate suppression of autoantigen-mediated B-cell receptor activation. *FcγRIIB* deficient mice had exacerbated autoimmune symptoms, and a partial restoration of functional *FcγRIIB* expression on B cells was sufficient to rescue mice from developing lupus-like disease^7,8^.

The involvement of FcγR in bone homeostasis has been studied. Activating, FcγRI, FcγRIIA, FcγRIII and FcγRIV, and inhibitory FcγR, FcγRIIB, are expressed on osteoclasts^9^. Binding of ICs that contain IgG to FcγR results in the activation of macrophages in synovial and cartilage layers. The activating FcγR directly induces severe cartilage destruction, but not bone erosion^10^. Arthritic mice lacking *FcγRI/II/III* demonstrated an increased level of joint inflammation. It was concluded that the absence of *FcγRI/II/III* drives joint inflammation, thereby indirectly causing bone resorption. Immunostaining revealed that the amount of RANKL-positive inflammatory cells was higher in the exudate of arthritic knee joints of FcγRI/II/III^−/−^ mice compared to controls. *FcγRIV* deletion in osteoclasts in a K/BxN serum transfer arthritis model decreased osteoclast differentiation without reducing the clinical signs of arthritis^9^. H-2^b^ mice deficient in *FcγRIIB* were susceptible to collagen-induced arthritis after immunization with native type II collagen^11^.

Our previous study indicated that mice with an *FcγRIIB* deletion resulted in SLE active disease at 6 months old. The *FcγRIIB*^−*/*−^ mice had cortical bone loss and decreased mechanical properties at 6 and 10 months old after the development of SLE or lupus disease^12^. The purpose of the present study was to determine the mechanism by which the absence of *FcγRIIB* affected cancellous bone turnover. Similar to the cortical bone phenotype, no change in cancellous bone volume was observed in 3-month-old *FcγRIIB*^−*/*−^ mice. However, 6 and 10 months old *FcγRIIB*^−*/*−^ males had cancellous bone loss due to elevated bone resorption without any change in bone formation. Deletion of *FcγRIIB* decreased cancellous bone volume in 10-, but not 6-month-old female knockouts. Serum TNFα level was increased in *FcγRIIB* knockouts. Etanercept, a TNFα inhibitor, increased cancellous bone volume and trabecular thickness in *FcγRIIB*^−*/*−^ mice. These data suggested that the absence of *FcγRIIB* induced inflammation and cancellous osteopenia in mice with lupus-like syndrome.

## Results

### *FcγRIIB* deletion did not affect cancellous bone in young adult mice

**Figure 1:**
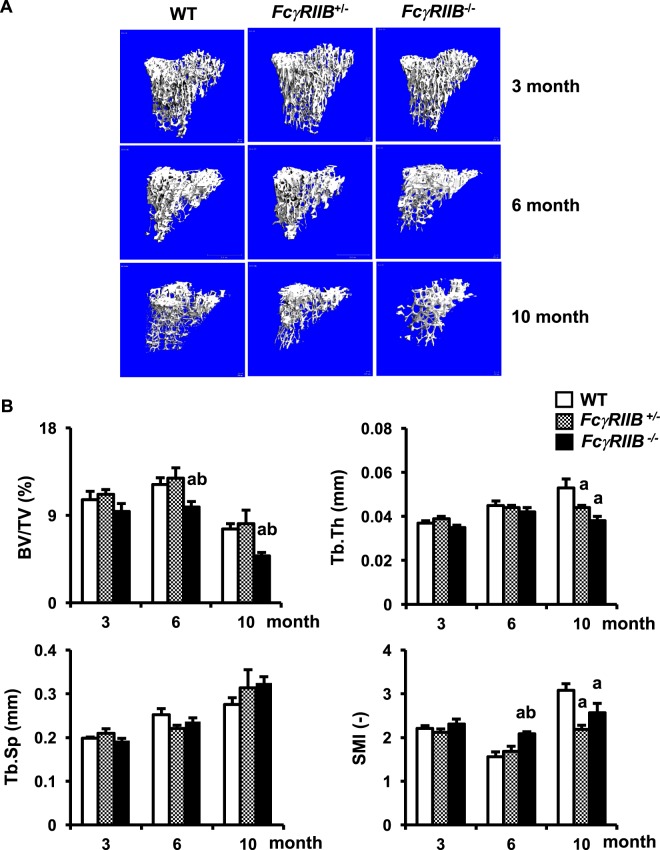
Absence of *FcγRIIB* induces cancellous bone loss in 6- and 10-month-old males. (**A**) Representative μCT images of the tibial cancellous bone from 3-, 6- and 10-month-old *FcγRIIB*^*+/*−^ and *FcγRIIB*^−*/*−^ males compared to their WT controls. (**B**) μCT analysis of the proximal tibial metaphysis. Results are mean ± SEM. ^a^*p* < 0.05 versus corresponding WT controls, and ^b^*p* < 0.05 versus corresponding *FcγRIIB*^*+/*−^ mice. BV/TV; bone volume per tissue volume, Tb.Th; trabecular thickness, Tb.Sp; trabecular separation and SMI; structural model index.

Our previous study indicated that 3-month-old *FcγRIIB*^−*/*−^ mice have a normal cortical bone phenotype^12^. To determine whether *FcγRIIB* deletion affected cancellous bone homeostasis in 3-month-old mice, we examine the skeletal phenotype of 3-month-old *FcγRIIB*^−*/*−^ and *FcγRIIB*^*+/*−^ males and their WT controls. μCT analysis revealed no difference in cancellous bone volume, trabecular thickness, trabecular number (data not shown), trabecular separation, or SMI between groups (Fig. 1A,B). Mice deficient in *FcγRIIB* on a C57BL/6 background developed a lupus-like autoimmunity at 6 months of age as indicated by an increased serum level of anti-dsDNA antibody and spleen B220^low^CD138^+^ plasma cells^12^. Therefore, our results indicated that *FcγRIIB* deletion did not affect skeletal homeostasis at 3 months of age when SLE disease was not active.

### 6- and 10-month-old *FcγRIIB*^−*/*−^ males had low cancellous bone volume due to increased osteoclast number

We examined whether the older *FcγRIIB*^−*/*−^ mice with active SLE had decreased cancellous bone. The μCT images and analysis of the proximal tibiae in 6- and 10-month-old *FcγRIIB*^−*/*−^ males are shown in Fig. 1A,B, respectively. Cancellous bone volume was decreased by 19% and structural model index was increased by 33% in 6-month-old *FcγRIIB*^−*/*−^ males. However, trabecular thickness, trabecular number (data not shown), and trabecular separation were not changed. Ten months old *FcγRIIB*^−*/*−^ males had decreased cancellous bone volume and trabecular thickness by 36% and 28%, respectively. Structural model index was decreased by 17%.

Histomorphometric analysis of the femur metaphysis showed a significant decrease in cancellous bone volume at 6 months of age in *FcγRIIB*^−*/*−^ males (Table 1). Trabecular thickness was decreased in knockouts. However, bone formation parameters, including mineral apposition rate, mineralizing surface, bone formation rate, osteoblast number, osteoid volume and osteoid thickness were not altered (Table 1). In contrast, osteoclast surface per bone surface, osteoclast number per tissue area and osteoclast number per bone perimeter were markedly increased at 6 months of age. Similar to the μCT analysis, the skeletal phenotype was more severe in older mice. *FcγRIIB* deletion dramatically decreased cancellous bone volume by 48%. Trabecular thickness, and trabecular number were decreased whereas trabecular separation was increased. Osteoclast surface per bone surface and osteoclast number per bone perimeter were increased compared to controls, leading to bone loss in *FcγRIIB*^−*/*−^ mice. There was no statistical significant difference between *FcγRIIB*^−*/*−^ and control mice in all indices of bone formation at 10 months of age.

**Table 1:** Histomorphometric analysis of femurs from 6- and 10-month-old *FcγRIIb*^−/−^ males and their control littermates.

Parameters	6-month-old		10-month-old	
	WT	*FcγRIIB*^−/−^	WT	*FcγRIIB*^−/−^
	(n = 7)	(n = 6)	(n = 7)	(n = 6)
BV/TV (%)	11.58 ± 1.17	7.76 ± 1.06*	8.66 ± 1.00	4.47 ± 0.32*
Tb.Th (μm)	44.93 ± 3.80	33.96 ± 2.09*	41.86 ± 3.10	29.62 ± 1.73*
Tb.N (/mm)	2.60 ± 0.22	2.42 ± 0.27	2.08 ± 0.17	1.51 ± 0.08*
Tb.Sp (μm)	356 ± 32	421 ± 67	462 ± 45	640 ± 32*
MS/BS (%)	20.64 ± 2.34	22.81 ± 2.54	14.88 ± 2.58	19.78 ± 2.78
MAR (μm/day)	1.14 ± 0.06	1.02 ± 0.04	1.28 ± 0.20	1.29 ± 0.40
BFR/BS (μm^3^/μm^2^/year)	85 ± 11	85 ± 11	70 ± 19	79 ± 11
BFR/BV (%/year)	415 ± 54	507 ± 73	357 ± 77	502 ± 54
BFR/TV (%/year)	54 ± 7	50 ± 9	27 ± 8	26 ± 4
Ob.S/BS (%)	7.10 ± 1.70	5.58 ± 1.42	2.16 ± 0.64	1.81 ± 0.61
N.Ob/T.Ar (/mm^2^)	31.03 ± 8.95	20.29 ± 5.70	6.61 ± 2.08	4.23 ± 1.30
N.Ob/B.Pm (/mm)	5.65 ± 1.27	4.33 ± 1.22	1.63 ± 0.55	1.47 ± 0.49
OV/TV (%)	0.023 ± 0.007	0.018 ± 0.008	0.006 ± 0.006	0.005 ± 0.002
OS/BS (%)	1.38 ± 0.39	1.10 ± 0.49	0.44 ± 0.39	0.54 ± 0.27
O.Th (μm)	2.18 ± 0.57	2.36 ± 0.77	0.95 ± 0.64	1.48 ± 0.68
Oc.S/BS (%)	0.31 ± 0.09	1.35 ± 0.47*	0.73 ± 0.23	1.87 ± 0.39*
N.Oc/T.Ar (/mm^2^)	0.58 ± 0.19	2.43 ± 0.81*	0.94 ± 0.30	1.67 ± 0.33
N.Oc/B.Pm (/mm)	0.11 ± 0.03	0.50 ± 0.17*	0.23 ± 0.07	0.54 ± 0.08*
ES/BS (%)	0.13 ± 0.08	0.73 ± 0.30	0.43 ± 0.16	0.85 ± 0.25

Results are mean ± SEM. *p < 0.05 compared to corresponding WT controls, unpaired *t*-test.

BV/TV; bone volume per tissue volume, Tb.Th; trabecular thickness, Tb.N; trabecular number, Tb.Sp; trabecular separation, MS/BS; mineralizing surface per bone surface, MAR; mineral apposition rate, BFR/BS; bone formation rate per bone surface, BFR/BV; bone formation rate per bone volume, BFR/TV; bone formation rate per tissue volume, Ob.S/BS; osteoblast surface per bone surface, N.Ob/T.Ar; osteoblast number per tissue area, N.Ob/B.Pm; osteoblast number per bone perimeter, OV/TV; osteoid volume per tissue volume, OS/BS; osteoid surface per bone surface, O.Th; osteoid thickness, Oc.S/BS; osteoclast surface per bone surface, N.Oc/T.Ar; osteoclast number per tissue area, N.Oc/B.Pm; osteoclast number per bone perimeter, and ES/BS; eroded surface per bone surface.

### *FcγRIIB*^−*/*−^ females were osteopenic at 10 months old

**Figure 2:**
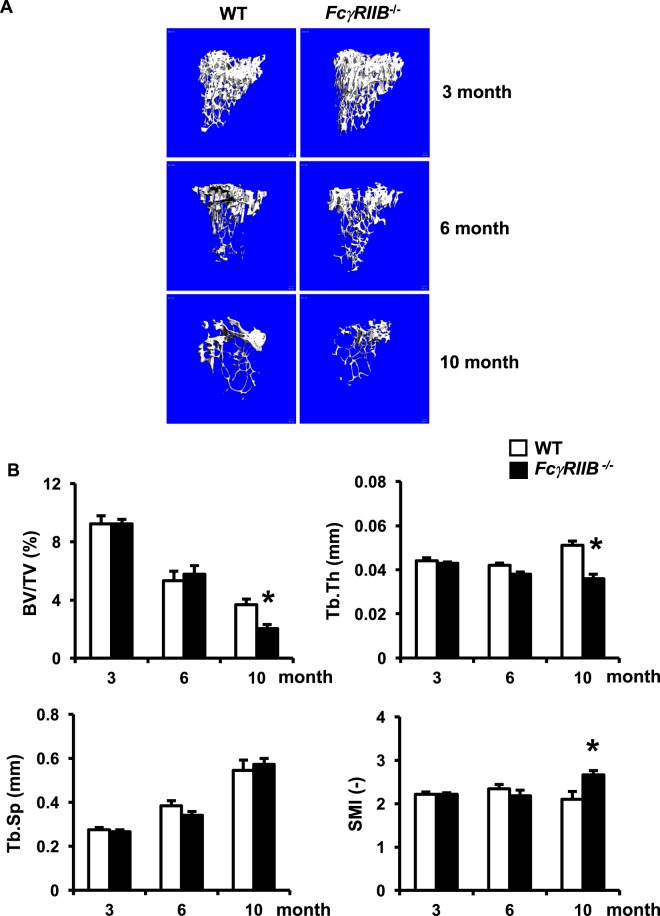
*FcγRIIB* deletion decreases cancellous bone volume in 10 months old females. (**A**) Representative μCT images of the tibial cancellous bone from 3-, 6- and 10-month-old *FcγRIIB*^−*/*−^ females and WT controls. (**B**) μCT analysis of the proximal tibial metaphysis. Results are mean ± SEM. *p < 0.05 versus corresponding WT controls. BV/TV; bone volume per tissue volume, Tb.Th; trabecular thickness, Tb.Sp; trabecular separation and SMI; structural model index.

To compare the skeletal phenotype observed in females to males, we performed μCT analysis in 6 and 10 months old *FcγRIIB*^−*/*−^ females. *FcγRIIB*^−*/*−^ females had a normal cancellous bone phenotype at 6 months of age (Fig. 2A,B). The μCT analysis indicated that cancellous bone microarchitecture did not change in 6-month-old *FcγRIIB*^−*/*−^ females. There was no change in cancellous bone volume, trabecular thickness, trabecular number (data not shown), trabecular separation, or SMI. However, 10-month-old *FcγRIIB*^−*/*−^ females were osteopenic (Fig. 2A,B). Cancellous bone volume was reduced due to decreased trabecular thickness, however, trabecular number (data not shown), and trabecular separation did not change. The higher SMI was observed in *FcγRIIB*^−*/*−^ females compared to WT controls.

### Increased bone resorption in *FcγRIIB*^−*/*−^ mice was due to increased osteoclastogenesis

**Figure 3:**
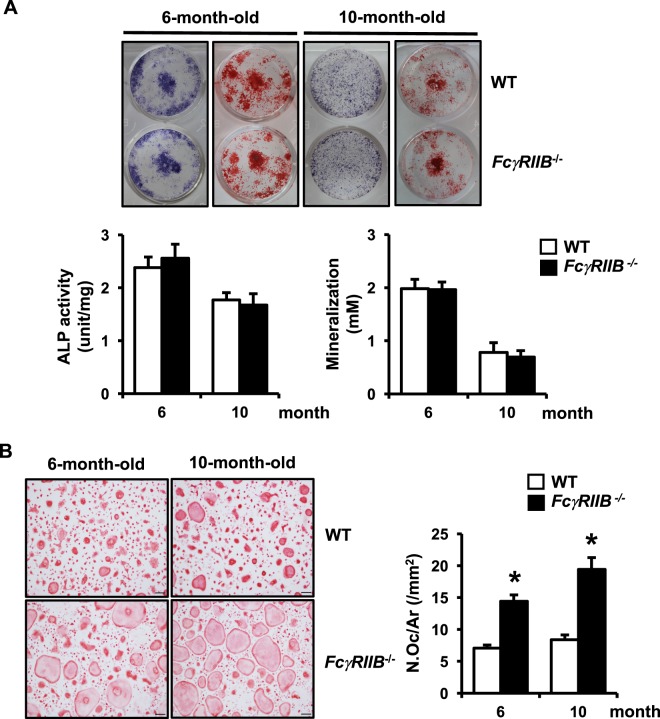
*FcγRIIB* deletion increases osteoclast, but not osteoblast differentiation. (**A**) ALP (left) and mineralized bone nodule (right) in osteoblasts derived from long bones of 6- and 10-month-old *FcγRIIB*^−*/*−^ males and WT controls. ALP activity (unit/mg) and mineralization or alizarin red concentration (mM) were quantified. (**B**) *FcγRIIB*^−*/*−^ and WT osteoclasts were generated on glass coverslips in the presence of M-CSF and RANKL (left). TRAP-positive spreading osteoclasts containing more than 5 nuclei per area were quantified by OsteoMeasure software, (OsteoMetrics, right). Results are mean ± SEM. *p < 0.05 versus corresponding WT controls. ALP; alkaline phosphatase, N.Oc; osteoclast number and Ar; area. Scale bar: 100 μm.

Histomorphometry indicated that *FcγRIIB* deletion led to increased osteoclast number without any change in osteoblast number in 6 and 10 months old males. To confirm that this deletion did not affect bone formation, we performed an *in vitro* assay using primary osteoblasts derived from long bones. ALP and mineralized bone nodules were similar in osteoblasts derived from *FcγRIIB*^−*/*−^ mice and their control littermates, indicating that deletion of *FcγRIIB* did not affect osteoblast differentiation or mineralization (Fig. 3A).

We determined whether the increased osteoclast number in *FcγRIIB*^−*/*−^ mice led to cancellous bone loss. An *in vitro* osteoclast differentiation assay was performed. Consistent with the increased bone resorption observed *in vivo*, deletion of *FcγRIIB* increased TRAP positive osteoclasts derived from bone marrow macrophages (BMMs) of both 6- and 10-month-old mice compared to WT controls (Fig. 3B). These data revealed the major role of *FcγRIIB* in osteoclast differentiation subsequent to SLE development.

**Figure 4:**
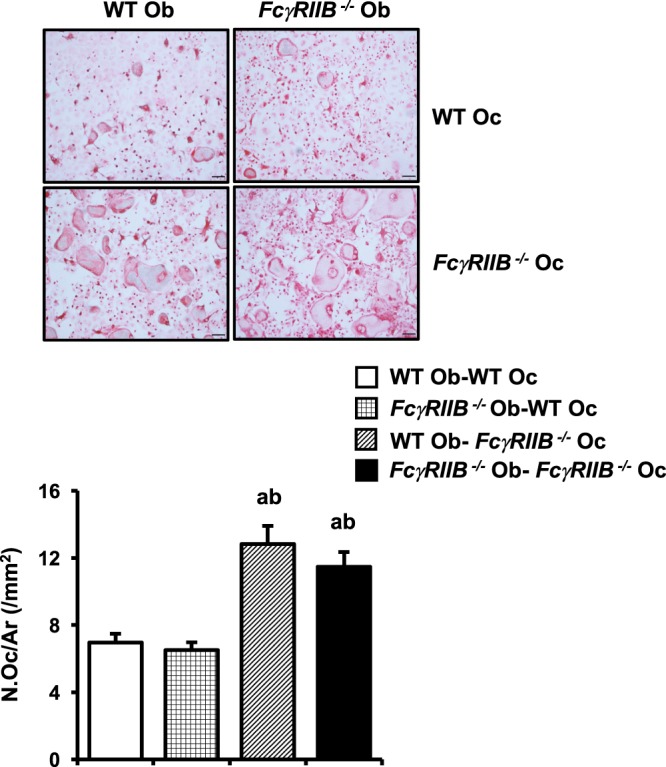
*FcγRIIB*^−*/*−^ osteoblasts had no effect on osteoclast differentiation. TRAP-positive spreading osteoclasts containing more than 5 nuclei per area from co-culture of either WT or *FcγRIIB*^−*/*−^ osteoblasts with WT or *FcγRIIB*^−*/*−^ BMMs. Results are mean ± SEM. ^a^*p* < 0.05 versus WT Ob-WT Oc, and ^b^*p* < 0.05 versus *FcγRIIB*^−*/*−^ Ob-WT Oc. Ob; osteoblasts, Oc; osteoclasts, N.Oc; osteoclast number and Ar; area. Scale bar: 100 μm.

To investigate whether the presence of *FcγRIIB*^−*/*−^ osteoblasts had a role in modulating osteoclast function, osteoblasts derived from *FcγRIIB*^−*/*−^ mice and WT controls were co-cultured with BMMs derived from either *FcγRIIB*^−*/*−^ mice or WT controls. Osteoclast number was increased when BMMs from *FcγRIIB*^−*/*−^ mice were co-cultured with osteoblasts from either *FcγRIIB*^−*/*−^ mice or WT (Fig. 4). We observed similar number of osteoclasts generated in co-cultures of WT BMMs with either *FcγRIIB*^−*/*−^ or WT osteoblasts. These data revealed that deletion of *FcγRIIB* in BMMs stimulated osteoclast differentiation.

### *FcγRIIB* regulates the expression of bone resorption genes

**Figure 5:**
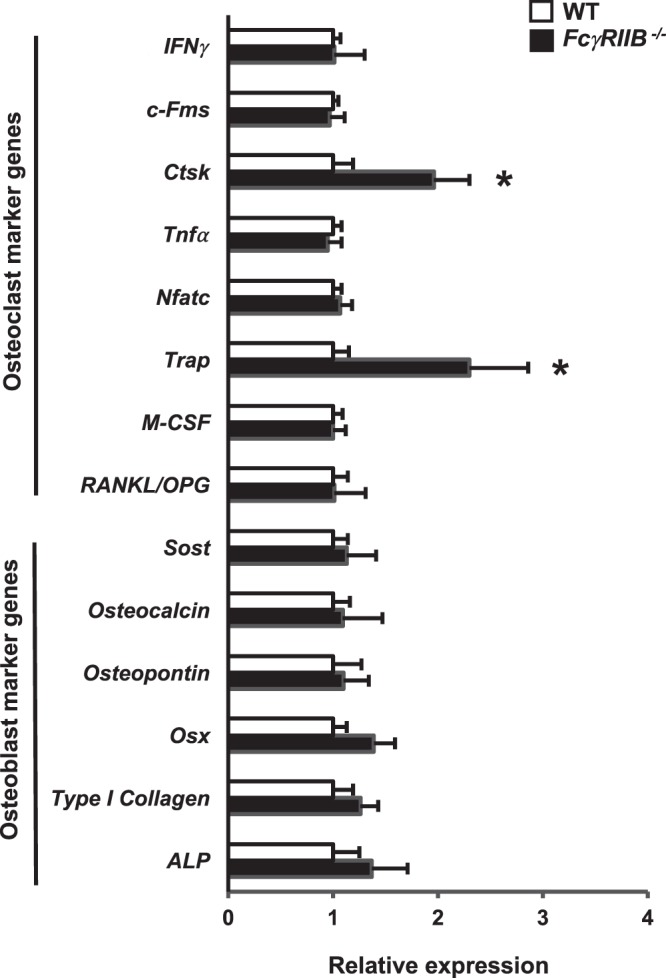
Absence of *FcγRIIB* upregulates osteoclast marker gene expression. qRT-PCR analysis of mRNA expression in the distal femur metaphysis from 10-month-old *FcγRIIB*^−*/*−^ males. Results are mean ± SEM. *p < 0.05 versus WT controls.

Deletion of *FcγRIIB* increased bone resorption without any effect on bone formation in both *in vivo* and *in vitro*. The skeletal phenotype was more pronounced in 10-month-old *FcγRIIB*^−*/*−^ males. We then selected these mice to determine whether *FcγRIIB* deletion affected any gene expression. qPCR analysis revealed that osteoblast marker genes, including *Alp*, *type I collagen*, *Osx*, *osteopontin*, *osteocalcin*, *and Sost* expression did not alter (Fig. 5). In contrast, deletion of *FcγRIIB* increased the mRNA levels for osteoclast marker genes, including *Trap*, *and Ctsk*. However, the *RANKL/OPG* ratio, *M-CSF*, *Nfatc1*, *Tnfα*, *c-Fms* and *IFNγ* mRNA levels were not affected in *FcγRIIB*^−*/*−^ mice.

### *FcγRIIB*^−*/*−^ mice produced higher TNFα serum level

**Figure 6:**
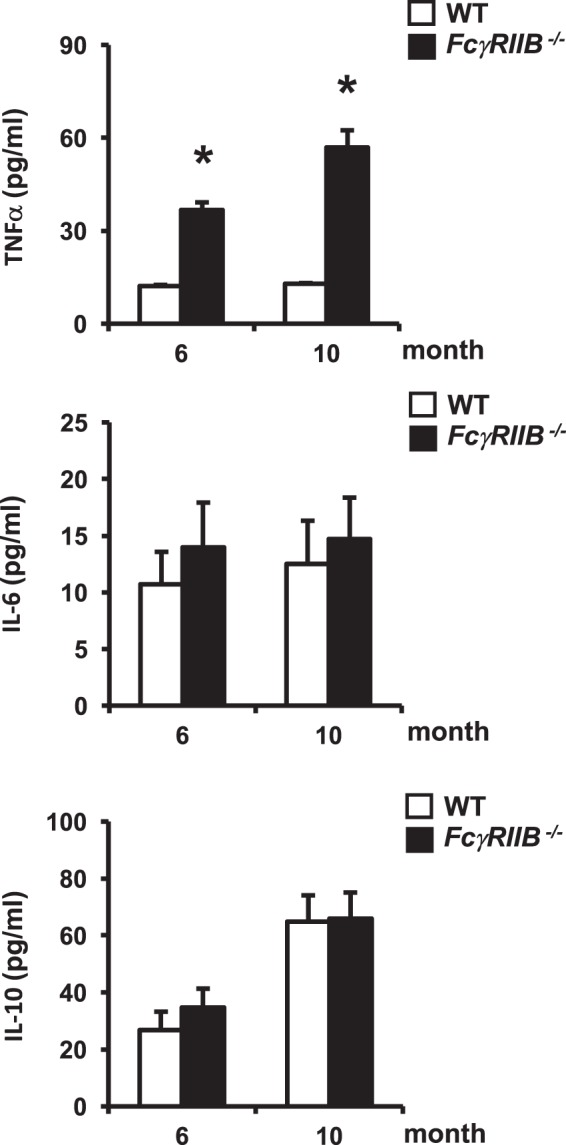
*FcγRIIB*^−*/*−^ mice had high serum levels of TNF-α. Serum levels of TNF-α, IL-6, and IL-10 in 6- and 10-month-old *FcγRIIB*^−*/*−^ males. Results are mean ± SEM. *p < 0.05 versus corresponding WT controls.

To investigate whether the absence of *FcγRIIB* increased inflammation, the serum levels of proinflammatory cytokines including TNFα, and IL-6 were determined in 6 and 10 months old *FcγRIIB*^−*/*−^ males and WT controls. *FcγRIIB* deletion increased the serum level of TNFα, whereas that of IL-6 was not affected (Fig. 6). The higher TNFα serum level was more pronounced in 10- compared to 6-month-old *FcγRIIB*^−*/*−^ mice. Serum IL-10, a potent anti-inflammatory cytokine, was also measured. We did not observe any change in the serum level of IL-10. We also determined whether the serum TNFα level was altered in 3 months old *FcγRIIB*^−*/*−^ males. There was no change in the serum TNFα level in 3-month-old *FcγRIIB*^−*/*−^ males compared to WT (5.52 ± 0.16 vs 5.98 ± 0.43 pg/ml). Our data indicated that the serum level of TNFα was increased following SLE development.

### TNFα antagonist prevents cancellous bone loss in *FcγRIIB*^−*/*−^ mice

**Figure 7:**
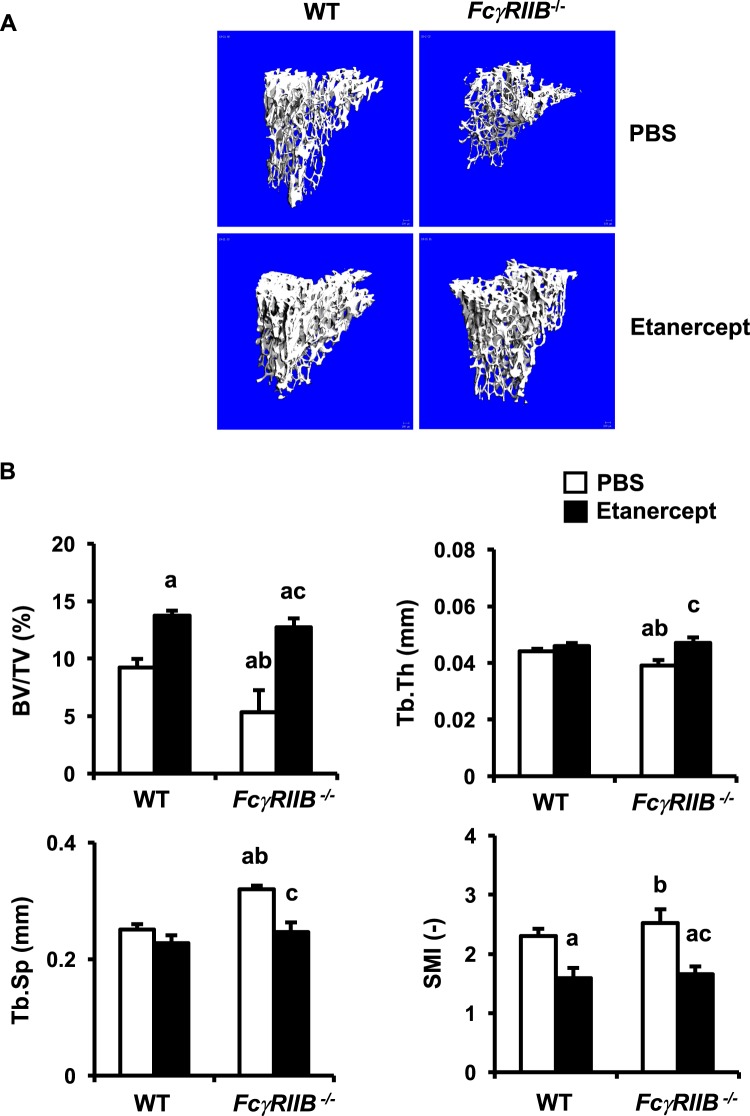
Blocking TNF-α prevents cancellous bone loss in *FcγRIIB*^−*/*−^ mice. (**A**) Representative μCT images of the tibial cancellous bone from WT and *FcγRIIB*^−*/*−^ males treated with either PBS or etanercept. (**B**) μCT analysis of the proximal tibial metaphysis. Results are mean ± SEM. ^a^*p* < 0.05 versus WT controls treated with PBS, ^b^*p* < 0.05 versus WT controls treated with etanercept, and ^c^*p* < 0.05 versus *FcγRIIB*^−*/*−^ mice treated with PBS.

We examined whether TNFα is a key factor that induced cancelllous bone loss in *FcγRIIB*^−*/*−^ mice. Etanercept, a recombinant human soluble fusion protein of TNFα type II receptor linked to Fc portion of IgG1, was used to block TNFα function. μCT data confirmed that 6-month-old *FcγRIIB*^−*/*−^ mice were osteopenic as mentioned earlier (Fig. 7). Inhibition of TNFα increased cancellous bone volume by 49 and 139% in WT and *FcγRIIB*^−*/*−^ mice, respectively. SMI was decreased by etanercept treatment in both *FcγRIIB*^−*/*−^ mice and WT controls. *FcγRIIB*^−*/*−^ mice treated with etanercept had increased trabecular thickness with a concomitant decrease in trabecular separation. Two-way ANOVA indicated no interaction between *FcγRIIB* deletion and etanercept on any parameter. These findings confirmed that TNFα mediated osteopenia in *FcγRIIB*^−*/*−^ mice and that blocking TNFα prevented cancellous bone loss.

## Discussion

SLE is an autoimmune disease characterized by B-cell hyperactivity and B-cell receptor signaling abnormalities. SLE patients have a high prevalence of osteoporotic fracture leading to increased morbidity. The role of *FcγRIIB* in SLE-associated bone loss is not completely understood. In this study, we determined whether deleting *FcγRIIB* affected cancellous bone phenotype. We found that cancellous bone volume was similar in 3-month-old *FcγRIIB*^−*/*−^ mice compared to WT controls. However, *FcγRIIB* deletion reduced cancellous bone volume at 6 and 10 months old in males. The cancellous osteopenia was associated with increased osteoclast number without any change in osteoblast number. The expressions of *Trap* and *Ctsk*, osteoclast marker genes, was upregulated in knockouts. Female *FcγRIIB*^−*/*−^ mice had decreased cancellous bone volume at 10, but not 6 months old. Serum TNF-α level, a proinflammatory cytokine, was increased in knockouts. Blocking TNF-α increased cancellous bone volume. Our data suggest that the absence of *FcγRIIB* results in cancellous bone loss due to inflammation-induced osteoclastic bone resorption in a murine model of spontaneous SLE-like syndrome.

The causes of bone loss in SLE are multifactorial, involving factors intrinsic to the disease itself, adverse effects of medication, and genetic factors. Moreover, proteinuria and renal dysfunction may aggravate vitamin D insufficiency, leading to bone loss in lupus nephritis patients. Low bone mass has an early onset in these patients, most likely due to systemic inflammation-induced increased osteoclastic bone resorption and reduced osteoblastic bone formation. Increased proinflammatory cytokines, including TNFα, IL-1, IL-6, and IL-17 levels might increase the production of RANKL^13^. In addition, activated synovial T cells and fibroblasts express RANKL and TNFα to stimulate osteoclast differentiation during inflammation. The inhibition of the BMP/Smad pathway suppresses osteoblast differentiation through activated NF-κB signaling in SLE patients^14^.

Glucocorticoid use is a well-known risk factor for osteoporosis and fracture. However, glucocorticoid-induced osteoporosis in patients with SLE is still controversial. It has been reported that patients recently diagnosed with SLE without glucocorticoid treatment have decreased BMD compared to age-matched healthy individuals^2^. The reduction of BMD is related to decreased serum osteocalcin and increased urinary pyridinoline, suggesting that the disease itself independent of glucocorticoid treatment may lead to bone loss^15^. These findings suggest that glucocorticoids are not the only mechanism responsible for low bone mass in patients with SLE.

Genetic factors contribute to SLE susceptibility and presumably have an important role in determining the etiology of the disease. FcγR regulates autoantibody and IC-induced inflammation. *FcγRIIB* is expressed on B-cells, mast cells, dendritic cells, neutrophils, macrophages, and osteoclasts^9,16^. Decreased expression of *FcγRIIB* on germinal center B-cells was associated with strain-specific susceptibility to autoimmune disease. *FcγRIIB*^−*/*−^ mice on the C57BL/6 background spontaneously developed hypergammaglobulinemia, autoimmune glomerulonephritis and IC-mediated SLE-like disease^7^. In contrast, BALB/c mice deficient in *FcγRIIB* did not develop autoimmunity. This finding confirmed our previous study where *FcγRIIB*^−*/*−^ mice on the C57BL/6 background developed SLE at 6 months of age^12^. The serum anti-dsDNA antibody levels and antibody-secreting B220^low^CD138^+^ plasma cells, markers for SLE, were increased in 6-, but not 3-month-old *FcγRIIB*^−*/*−^ mice compared to WT controls. *Fcγ*RIIB-expressing retrovirus restored tolerance and prevented autoimmune disease in *FcγRIIB*^−*/*−^ mice^8^. The inhibitory role of *FcγRIIB* in the development of autoimmunity is supported by the evidence of SLE-associated *FcγRIIB* polymorphism in human^17^. In contrast, genetically deleting the activating *FcγRs* did not develop spontaneous autoimmune diseases in mice^18^. In addition, *FcγR* deletion did not have any effect on cancellous bone volume and osteoclast number^19^.

Despite extensive studies, genetic factors contributing to the increased bone loss in patients with SLE were not completely understood. The absence of activating *FcγRs*, *FcγRI*, *FcγRIII or FcγRIV* did not affect osteoclast differentiation and bone homeostasis at steady state^9^. No differences in cancellous bone volume, trabecular number, trabecular thickness, trabecular separation, or osteoclast number were observed among strains of knockouts. The role of FcγRI, FcγRIII, and FcγRIV on bone resorption during inflammatory arthritis was investigated. Lack of *FcγRIII* attenuated arthritis development and decreased inflammatory infiltration and cartilage destruction whereas deletion of *FcγRI* had no effect on arthritis after transfer of K/BxN serum^20^. *FcγRIV*-deficient mice had a smaller area of inflammatory infiltrate and bone erosion^9^.

Our studies indicated that the inactivation of *FcγRIIB* resulted in cancellous osteopenia due to increased osteoclastic bone resorption following SLE development at 6 months of age. These data coincided with the lower lumbar spine and hip BMD in patients with SLE^15,21^. Ten-month-old *FcγRIIB*^−*/*−^ mice had a substantial reduction in cancellous bone volume compared to younger mice, suggesting more severe bone loss with longer SLE disease duration. It has been reported that the inhibitory effect of *FcγRIIB* on osteoclast differentiation was mediated through IgGs^22^. IgG1 has a higher affinity for FcγRIIB and exhibits the strongest level of FcγRIIB-induced negative regulation^3^. The level of IgGs in *FcγRIIB*^−*/*−^ mice increased with age^22^. It is possible that the IgGs produced in *FcγRIIB*^−*/*−^ mice induces proinflammatory response during autoimmune disease, leading to cancellous bone loss.

Although the relationship between inflammatory cytokines and bone loss in patients with SLE remains unclear, increased osteoclast differentiation induced by proinflammatory cytokines is thought to be the cause of bone resorption in these patients. The inhibitory *FcγRIIB* is expressed on mast cells and macrophages which have the capacity to trigger strong proinflammatory responses. Patients with SLE are unable to perform IC clearance. These ICs can induce the production of cytokines, including IL-6, TNF-α and IL-10 by macrophages and dendritic cells^23,24^. Enhanced production of TNF-α in 6- and 10-month-old *FcγRIIB*^−*/*−^ mice in our study coincides with the high serum level of TNF-α in the patients with active SLE disease^25^. However, serum IL-6 and IL-10 levels were unaltered. The role of *FcγRIIB* in the release of proinflammatory mediator was demonstrated by enhanced macrophage responses in *FcγRIIB*^−*/*−^ mice with collagen-induced arthritis and IC-mediated alveolitis models^11,26^. TNF-α antagonist, etanercept, is widely used for the treatment of inflammatory diseases, including rheumatoid arthritis, axial spondyloarthritis, psoriatic arthritis, and plaque psoriasis^27^. Our study indicated that etanercept rescued the skeletal phenotype of *FcγRIIB*^−*/*−^ mice, indicating that TNF-α played a major role in cortical and cancellous bone loss in these mice.

MRL/lpr and BXSB/MpJ-Yaa mice are promising models of osteoporosis in murine lupus. It was found that MRL/lpr mice were osteopenic due to decreased bone formation^28^. The BXSB/MpJ-Yaa mice have Yaa mutation on Y chromosome, and male mice therefore develop severe SLE disease. Three months old BXSB/MpJ-Yaa males had a normal skeletal phenotype^29^. But osteopenia developed at 6 months of age. TRAP positive osteoclasts were increased without any change in osteocalcin positive cells, indicating increased bone resorption with normal bone formation. MRL/lpr mice have biphasic increase in circulating level and renal expression of TNF-α^30^. The serum TNF-α level was peak in neonatal mice, normalized by 2 months of age and continuously increased again afterward. The renal TNF-α expression was detected in neonatal mice and dramatically decreased within 2 weeks. However, the expression was increased with progressive lupus nephritis in aged mice.

Deleting *FcγRIIB* increased *Ctsk* and *Trap* mRNA expression, suggesting elevated bone resorption. In addition to Ctsk’s known function in osteoclasts, Ctsk may stimulate bone resorption through immune cell-mediated osteoclast activation. *Ctsk* deficiency reduced the proinflammatory cytokine expression in rheumatoid arthritis^31^. Trap is essential for skeletal development, bone mineralization, collagen metabolism, and cytokine production by dendritic cells and macrophages. Mice lacking *Trap* had an osteopetrotic phenotype, reduced resorptive activity during endochondral ossification, premature mineralization of epiphyseal cartilage, and shortened bones^32^.

Taken together, this study establishes the role of *FcγRIIB* on cancellous bone homeostasis as a link between SLE disease and osteoclast formation. Absence of *FcγRIIB* induces inflammation by enhancing TNF-α and increases osteoclastic bone resorption, resulting in cancellous bone loss in mice with active SLE disease. Therefore, FcγRIIB is an important candidate as a therapeutic target for autoimmune diseases and osteoporosis.

## Materials and Methods

### Experimental design

Male *FcγRIIB*^−*/*−^ mice on C57BL/6 background provided by Dr. Silvia Bolland (NIAID, NIH, Maryland, USA) were housed at the Faculty of Medicine, Chulalongkorn University. The experimental protocol was approved by the Institutional Animal Care and Use Committee at the Faculty of Medicine, Chulalongkorn University in accordance with the Guide for the Care and Use of Laboratory Animals (eight edition), National Research Council. The mice were maintained at room temperature and housed on a 12 h light and 12 h dark cycle during the study. Standard mouse chow (C.P. Mice Feed, Perfect Companion Group Co., Ltd., Thailand) and water were provided *ad libitum* to all mice. Male *FcγRIIB*^−*/*−^ mice were crossed with C57BL/6 females purchased from the National Laboratory Animal Center, Mahidol University, Thailand to generate heterozygotes. Male and female *FcγRIIB*^*+/*−^ mice were crossed to obtain *FcγRIIB*^*+/*−^, *FcγRIIB*^−*/*−^, and wild type (WT) control mice. The mice were genotyped using PCR (95 °C for 15 s, 60 °C for 30 s, and 72 °C for 30 s for 35 cycles) using 3 primers: 5′-AAGGCTGTGGTCAAACTCGAGCC-3′, 5′-CTCGTGCTTTACGGTATCGCC-3′ and 5′-TTGACTGTGGCCTTAAACGTGTAG-3′.

The mice received double fluorochrome labeling with 20 mg/kg calcein (Sigma, St. Louis, MO, USA) to label mineralizing bone. The interlabel periods were 7 and 8 days for 6 and 10 months old *FcγRIIB*^−*/*−^ mice and WT controls, respectively. Three-, 6- and 10 months old mice were anesthetized with isoflurane and sacrificed by cervical dislocation. The left tibiae and femurs were fixed in 70% alcohol for microcomputed tomography (*μ*CT) and histomorphometric analyses, respectively. The right femurs were removed and kept at −80 °C for RNA isolation and qPCR analysis.

For TNF-α inhibitor administration, 6 months old *FcγRIIB*^−*/*−^ males and their WT controls were subcutaneously injected with either PBS or 25 mg/kg etanercept (Wyeth, New Jersy, USA) twice a week for 8 weeks. This dose is an intermediate dose for mice^33^. At the end of experiment, left tibiae were removed and fixed in 70% alcohol for *μ*CT analysis

### µCT analysis

µCT was used for nondestructive 3-dimensional evaluation of the bone microarchitecture using a µCT35 scanner (Scanco Medical AG, Bassersdorf, Switzerland) according to standard guidelines^34^. This technique quantitatively assesses cancellous and cortical bone morphology which provides information about the amount of bone. The bone samples were scanned at a voxel size of 7 µm, 50 kVp, 144 µA and 800 ms integration time. The machine was set at a threshold of 220 to distinguish bone from soft tissues. Four hundred and sixty four transverse slices of the cancellous bone at the proximal tibial metaphysis were scanned. Cancellous bone was assessed in 300 transverse slices to determine bone volume (BV/TV, %), trabecular thickness (Tb.Th, mm), trabecular number (Tb.N, /mm), trabecular separation (Tb.Sp, mm), and structural model index (SMI, -).

### Histomorphometry

The left femurs were dehydrated in graded acetone, infiltrated and embedded undecalcified in methyl methacrylate. Longitudinal sections (4 µm thick) were cut with a microtome (Leica 2065). A section was left unstained for determining the dynamic parameters including mineralizing surface per bone surface (MS/BS, %), mineral apposition rate (MAR, μm/day), and bone formation rate adjusted for bone surface (BFR/BS, µm^3^/µm^2^/year), bone volume (BFR/BV, %/year) and tissue volume (BFR/TV, %/year). A consecutive section was stained with toluidine blue for cell-based measurements, including osteoblast surface per bone surface (Ob.S/BS, %), osteoblast number per bone perimeter (N.Ob/B.Pm, /mm), osteoblast number per tissue area (N.Ob/T.Ar, /mm^2^), osteoid thickness (O.Th, µm), osteoid surface per bone surface (OS/BS, %), osteoid volume per tissue volume (OV/TV, %), osteoclast surface per bone surface (Oc.S/BS, %), osteoclast number per bone perimeter (N.Oc/B.Pm, /mm), osteoclast number per tissue area (N.Oc/T.Ar, /mm^2^), and eroded surface per bone surface (ES/BS, %). Histomorphometric data were obtained using the OsteoMeasure System (OsteoMetrics, Inc., Atlanta, GA, USA). All parameters were reported using standardized nomenclature^35^.

### Osteoblast differentiation

Primary osteoblasts derived from the long bones of *FcγRIIB*^−*/*−^ males and their control littermates were prepared as previously described^36^. The cells were cultured in differentiation medium containing α-MEM supplemented with 10% FBS, 100 unit/ml penicillin and 100 μg/ml streptomycin, 10 μM dexamethasone, 5 mM β-glycerophosphate, and 50 μg/ml ascorbic acid. Osteoblasts were fixed with 3.7% formaldehyde, and stained for ALP and mineralized bone nodules with Fast Blue RR (Sigma, St. Louis, MO, USA), and 2% alizarin red (Sigma, St. Louis, MO, USA) on days 7 and 21, respectively. ALP activity was quantified as previously described^37^. Mineralization was measured by extraction of calcified mineral stained with alizarin red using 10% cetylpyridinium chloride in 10 mM sodium phosphate (pH 7)^36^. The concentration of alizarin red (mM) after destaining was determined.

### Osteoclast differentiation

Bone marrow cells were cultured in α-MEM containing 10% FBS, 100 unit/ml penicillin and 100 μg/ml streptomycin for 24 h to obtain BMMs. The BMMs were then cultured on coverslips in α-MEM containing 20 ng/ml M-CSF (R&D Systems, Inc., MN, USA) for 2 days and in the same medium containing 20 ng/ml M-CSF, and 3.3 ng/ml RANKL (R&D Systems, Inc., MN, USA) for an additional 6 days. TRAP-positive spreading osteoclasts containing more than 5 nuclei were quantified using OsteoMeasure software.

For co-culture studies, osteoblasts derived from the long bones of *FcγRIIB*^−*/*−^ males and their WT controls were cultured in α-MEM containing 10% FBS, 100 unit/ml penicillin and 100 μg/ml streptomycin for 24 h. BMMs from either *FcγRIIB*^−*/*−^ mice or WT controls were added and cultured in α-MEM containing 10^−6^ M prostaglandin E_2_ (Merck Millipore, Murlington, MA, USA) and 10^−8^ M 1,25-dihydroxyvitamin D_3_ (Merck Millipore, Murlington, MA, USA) for 4 days. TRAP-positive spreading osteoclasts containing more than 5 nuclei were counted using OsteoMeasure software.

### qPCR analysis

The right femur distal metaphyses were pulverized in liquid nitrogen. Total RNA was isolated using Trizol reagent (Invitrogen, Carlsbad, CA, USA) according to the manufacturer’s protocol. The samples were purified using an RNeasy Mini kit (Qiagen, Hilden, Germany). cDNA was synthesized using SuperScript VILO (Invitrogen, Carlsbad, CA, USA). The qPCR was performed at 60 °C for 40 cycles using CFX96^TM^ Optics Module (Bio-Rad, CA, USA) and GAPDH was used as an internal control for quantification. The primer sequences are listed in Supplementary Table S1.

### Serum cytokines

Serum cytokines, TNF-α, IL-6, and IL-10 were measured by ELISA according to the manufacturer’s protocol (ThermoFisher Scientific, Waltham, MA, USA).

### Statistical analysis

All data are expressed as mean ± SEM. The unpaired Student’s t-test was used to compare the differences between 2 groups. The significance of differences between 3 groups was analyzed using one-way ANOVA followed by Fisher’s protected least significant difference test. Interactions between *FcγRIIB* deletion and etanercept were determined by two-way ANOVA. Differences were defined as significant at *p* < 0.05.

## Supplementary information

Supplementary Information

## Data Availability

All data are available from the corresponding author upon request.
