# Supplementary material for: Lupus-like Disease in FcγRIIB−/− Mice Induces Osteopenia
Source: Sci Rep. 2019 Nov 22;9:17342. doi: 10.1038/s41598-019-53963-z (PMC6874658; doi:10.1038/s41598-019-53963-z)
Supplement: Supplementary file 1 — Supplementary Information [file 41598_2019_53963_MOESM1_ESM.pdf]

Lupus-like Disease in *FcγRIIB*$^{-/-}$ Mice Induces Osteopenia

Peerapat Visitchanakun[1,2], Worasit Saiworn[1,2], Prapaporn Jongwattanapisan[3], Asada Leelahavanichkul[4], Prapaporn Pisitkun[5], Sutada Lotinun[1,2]*

[1]Department of Physiology, Faculty of Dentistry, Chulalongkorn University, Bangkok, Thailand
[2]Skeletal Disorders Research Unit, Faculty of Dentistry, Chulalongkorn University, Bangkok, Thailand
[3]Department of Veterinary Medicine, Faculty of Veterinary Science, Chulalongkorn University, Bangkok, Thailand
[4]Division of Immunology, Department of Microbiology, Faculty of Medicine, Chulalongkorn University, Bangkok, Thailand
[5]Division of Allergy, Immunology, and Rheumatology, Department of Medicine, Faculty of Medicine, Ramathibodi Hospital, Mahidol University, Bangkok, Thailand

*Address correspondence to:

Sutada Lotinun, Ph.D.
Department of Physiology
Faculty of Dentistry
Chulalongkorn University
Bangkok, Thailand
Tel: +662 218-8694
Fax: +662 218-8691
Email: sutada.l@chula.ac.th

Table S1 Primer sequences for qPCR analysis

Gene	Forward primer	Reverse primer
Alp	CTTGACTGTGGTTACTGCTGATCA	GTATCCACCGAATGTGAAAACGT
Type I collagen	CCCAAGGAAAAGAAGCACGTC	ACATTAGGCGCAGGAAGGTCA
Osx	CCCTTCTCAAGCACCAATGG	AAGGGTGGGTAGTCATTTGCATA
Osteopontin	CTCCAATCGTCCCTACAGTCG	CCAAGCTATCACCTCGGCC
Osteocalcin	GCTGCCCTAAAGCCAAACTCT	AGAGGACAGGGAGGATCAAGTTC
Sost	ATCATTTCCAGACACCTCTTAC	ATGTGCTTCTGTTACAAACGCTC
RANKL	GGAAGCGTACCTACAGACTATC	CTCCCTCCTTTCATCAGGTTAT
OPG	AAGAGCAAACCTTCCAGCTGC	CACGCTGCTTTCACAGAGGTC
M-CSF	ACCTGTTTCCCAAGAAGAGAGCCT	AGCTGTCAACACAAGCAGCCAAAG
Trap	GATCCCTCTGTGCGACATCA	CCAGGGAGTCCTCAGATCCA
Nfatc1	AGGCTGGTCTTCCGAGTTCA	ACCGCTGGGAACACTCGAT
Tnfα	TTGTCTACTCCCAGGTTCTCT	GAGGTTGACTTTCTCCTGGTATG
Ctsk	AGGCATTGACTCTGAAGATGCT	TCCCCACAGGAATCTCTCTG
c-Fms	TGGCATCTGGCTTAAGGTGAA	GAATCCGCACCAGCTTGCTA
IFNγ	AAATCCTGCAGAGCCAGATTAT	GCTGTTGCTGAAGAAGGTAGTA
GAPDH	TGCACCACCAACTGCTTAG	GGATGCAGGGATGATGTTC
